# Diversification of PAR signaling through receptor crosstalk

**DOI:** 10.1186/s11658-022-00382-0

**Published:** 2022-09-10

**Authors:** Irene Lee-Rivera, Edith López, Ana María López-Colomé

**Affiliations:** Department of Molecular Neuropathology, Instituto de Fisiología Celular, UNAM, Apartado Postal 70-253, Ciudad Universitaria, Mexico City, CdMx, Mexico

**Keywords:** Receptor dimerization, GPCR transactivation, Receptor cofactoring, Receptor signalling crosstalk

## Abstract

Protease activated receptors (PARs) are among the first receptors shown to transactivate other receptors: noticeably, these interactions are not limited to members of the same family, but involve receptors as diverse as receptor kinases, prostanoid receptors, purinergic receptors and ionic channels among others. In this review, we will focus on the evidence for PAR interactions with members of their own family, as well as with other types of receptors. We will discuss recent evidence as well as what we consider as emerging areas to explore; from the signalling pathways triggered, to the physiological and pathological relevance of these interactions, since this additional level of molecular cross-talk between receptors and signaling pathways is only beginning to be explored and represents a novel mechanism providing diversity to receptor function and play important roles in physiology and disease.

**Supplementary Information:**

The online version contains supplementary material available at 10.1186/s11658-022-00382-0.

## Background

Protease activated receptors (PARs) are a small family of G protein-coupled receptors (GPCR) that mediate the cellular effects of serine proteases, mainly of those involved in the coagulation process. Together, the coagulation cascade and protease-activated receptors, provide an elegant mechanism linking tissue injury, to cellular responses, including cell proliferation, migration, and the release of inflammatory mediators, among others [1].

In addition to coagulation, PARs have been shown to play major roles in the cardiovascular, nervous, and gastric, systems, as well as in the lung, airways, skin, and the immune system (for review see [1]). In the vascular system, these receptors induce endothelium-dependent relaxation and contraction, therefore contributing to the regulation of vascular tone [2]. Additionally, PAR activation on endothelial cells induces a proinflammatory phenotype, increases vascular permeability, and promotes the exposure of proteins and the secretion of cytokines. Overall, this pathway mediates important processes including the local accumulation of platelets and leukocytes, therefore contributing to the vascular consequences of sepsis and diseases such as acute lung injury and acute respiratory distress syndrome [2]. Moreover, PARs also contribute to the hypertrophy of vascular smooth muscle cells and the production of extracellular matrix, involved in the pathophysiology of atherosclerosis and hypertension [2].

PARs are activated by a subset of serine proteases, most notably thrombin, but also by trypsin, factor Xa, plasmin, kallikreins, activated protein C (aPC), matrix metalloproteinases, tryptase and matriptase, neutrophil elastase, and neutrophil proteinase-3 can also activate these receptors [3]. PARs exhibit a unique mechanism of proteolytic activation: The cleavage of the N-terminus, exposes a new N-terminal peptide, which in turn binds to a conserved region on the second extracellular loop of the receptor, forming a “tethered ligand” that triggers intracellular signalling [4]. Although these proteases may sever distinct bonds within the N-terminus, the exact site depends on the membrane environment and cofactors. The use of these “alternative cleavage sites” provides diversity to PAR receptor signaling [4]. Importantly, downstream signalling can also be affected by the posttranslational modification of receptors, or the cofactors involved [3, 5].

The prototypical PAR receptor, PAR1, was the first member of this family to be identified, 30 years ago [6]. Subsequently, three other family members were identified (PAR2, PAR3, and PAR4) by homology screening of complementary DNA libraries [7–9]. The activation of PARs activates multiple signalling pathways, since they are coupled to G_αq_, G_α12,13_, G_αi_, and G_βγ_. Besides the differences arising from differential cleavage sites, the activation of specific G_α_-subunits triggers signalling pathways that vary according to tissue, and cell context, as well as the availability of co-receptors and regulate a variety of cellular functions (Additional file 1: Table S1).

Although G-protein-coupled receptors (GPCRs) were first proposed to function as monomeric units, they have been shown to dimerize, either with themselves or with other types of receptors [10]. This discovery has unveiled a novel level of molecular cross-talk between signalling pathways, and it represents a new mechanism for the regulation of GPCR function under both physiological and pathological conditions. The transactivation and multimerization of distinct GPCRs is an under-studied field, however it has an enormous potential to unveil a new generation of pharmacological targets for which no gene has been identified so far. On this line, PARs have shown a wide capacity for interaction and receptor crosstalk. PARs are able to form both homodimers and heterodimers among the members of their own family, which diversifies receptor function and signalling [11]. Moreover, the transactivation and co-factoring of PARs with other GPCRs, or with a wide variety of membrane proteins including receptor kinases or ionic channels, might have important physiological and pathological repercussions, which have only marginally been addressed [11, 12]. This field is in the verge of a major expansion, and this review is aimed to inspire future research. All four PARs have been shown to assemble into heterodimers, the best-defined being PAR1-PAR4 [13] and PAR1-PAR2 [14], which play an important role in the treatment of arterial thrombosis and sepsis, respectively [2]. The physiological relevance of PAR homodimerization has been difficult to assess, in spite of evidence for PAR1, 2 and 4 assembly into homodimers. It is noticeable that the conditions for this association are not fully understood. Additionally, we will discuss the transactivation of PARs by distinct receptors, particularly by receptor-tyrosine-kinases and receptor-serine/threonine-kinases. We will also underline signalling crosstalk in which transactivation has been shown, but on which the direct interaction between receptors has not been previously addressed. We believe that due to the impact of these associations on cellular physiology, elucidation of these interactions may represent an important opportunity to better understand cell physiology and discover future therapeutic approaches.

## PAR homodimers

The unusual activation mechanism of PARs has led to the design of a variety of ligands aimed to analyse their function. Among these, a considerable amount of work has been addressed by means of synthetic peptides that mimic the sequence generated by the cleavage of the receptor N-terminus carried by proteases, as well as small molecule agonists and peptidomimetic modulators [15]. The use of these agonists has allowed the unravelling of signaling pathways, triggered by these receptors in different contexts. However, the use of these peptides does not provide insight on the structural requirements for PAR activation. It is also noticeable, that the functional responses triggered by these peptides are not necessarily the same as those triggered by thrombin [16] and in some instances even have been shown to differ [17]. This suggests that the interaction, and possibly the conformational changes induced by the binding of the tethered ligand within the receptors, are more complex than those triggered by the isolated sequence of the agonist peptide. Moreover, thrombin has been shown to interact with two receptors at once, and activate both simultaneously, as in the coactivation of PAR1 or PAR3 and PAR4, that will be discussed below [18, 19].

Additionally, studies using mutations on the extracellular regions of PAR1 or PAR2 have demonstrated differences between the intra- versus inter-molecular mechanisms of PAR activation [20, 21]. Therefore, even though there is little information regarding the physiological consequences of PAR homodimerization, there is evidence for their association, which represents a major opportunity for further studies. The first work suggesting that PAR1 self-associates, giving rise to homodimers, came from a study using mutations on either the tethered ligand domain or on signalling relevant sites [22]. By means of co-transfection assays, Chen et al. showed that PAR1 signalling-defective mutants were able to activate mutants on the tethered ligand domain, thereby demonstrating that PAR1 can bind intermolecularly to activate an adjacent PAR1. The self-association of PAR2 was also shown using this approach, in addition to the coprecipitation of differentially epitope-tagged receptors [23]. Another method for assessing receptor association is bioluminescence resonance energy transfer (BRET), which has been used to demonstrate both, the direct interaction among constitutive PAR1 homodimers and its localization at the cell surface [24], as well as the formation of PAR4 homodimers [25]. It is of particular interest that PAR4 dimer interface has been mapped to hydrophobic residues in transmembrane helix 4 [25]; notably, these residues are the same ones that interact with PAR1 to form a heterodimer [25, 26]. Mutations that interrupt dimer association, also reduce PAR4 agonist- induced calcium mobilization, which associates the loss of dimer formation to the lack of PAR4 signaling [25]. Future studies must analyze the physiological consequences of PAR homodimerizaton, and furthermore, their function in the context of monomer activation or potential heterodimer interactions. Another relevant question to be considered, will be the differences in affinity and activation rates for monomer, homodimers and heterodimers, as well as, the dynamics of these interactions and their physio-pathological impact.

## PAR heterodimers

Evidence for PAR heterodimerization is abundant, and its physiological relevance has been well documented. Nakanishi- Matsui et al. proposed for the first time the concept of “cofactoring” in order to explain the induction of mouse platelet activation by the joint activation of PAR3-PAR4 dimer, since PAR3 lacks an intracellular signaling domain, and PAR4 shows low affinity for thrombin [18]. PAR1-PAR4 plays a similar role in human platelets, but as will be discussed further down, responses elicited by these interactions are not simple and might play specific roles in cell physiology. In this sense, PAR heterodimerization is more akin to the concept of “transactivation”, which refers to “the activation of one GPCR that leads rapidly and in the absence of de novo protein synthesis, to the activation and cytosolic generation of the immediate down-stream signaling of a second cell surface receptor” [27]. PAR transactivation diversifies receptor functions by triggering distinct signaling pathways involved in different cellular responses, or it can aid cellular trafficking of the receptors from the ER to the cell surface, as is the case for the PAR2-PAR4 duplet [28].

### PAR4 heterodimers

PAR4 heterodimers have been mostly analyzed in platelets, in which PARs play a crucial role in ATP secretion and aggregation. Mouse platelets do not express PAR1, and thrombin exerts it effects by activating mPAR3 and mPAR4. As mentioned above, mPAR3 lacks a cytoplasmic signaling domain [7]; on the other hand, mPAR4 has low affinity for thrombin, and the co-expression of mPAR3 has been shown to enhance thrombin effect by facilitating its cleavage, therefore acting as a cofactor for mPAR4 [18]. In mPAR4 deficient platelets, thrombin failure to elicit platelet activation or aggregation, confirmed that mPAR3 does not promote trans-membrane signaling on its own, but functions as a cofactor to aid mPAR4 cleavage by thrombin and its subsequent activation [18]. The direct interaction of mPAR3 with mPAR4 has been confirmed by X-ray crystallography studies using N-terminal fragments of murine PAR3 and PAR4 bound to thrombin [29]. BRET has also been used to demonstrate that murine PAR3 and PAR4 form constitutive heterodimers when expressed in HEK293 cells [30].

**Fig. 1:**
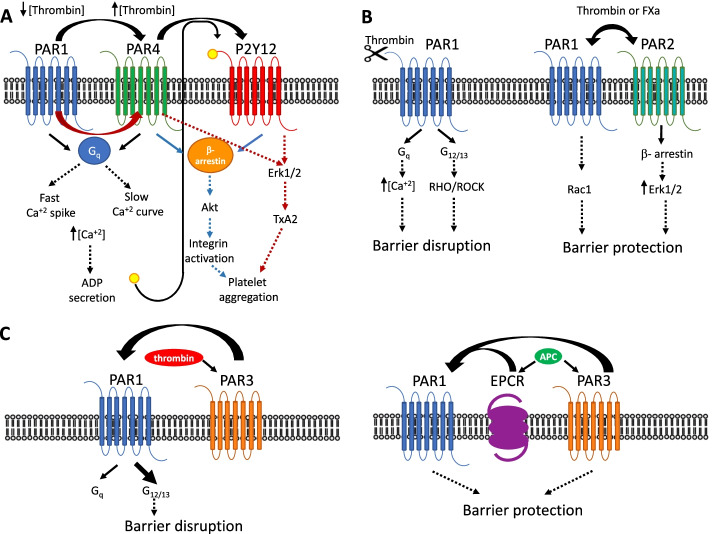
PAR heterodimerization. **A** PAR1/PAR4—P2Y12. PAR1-PAR4 heterodimer is required for thrombin response within a wide concentration range. Activation of the G_αq_ pathway (black arrows), promotes ADP secretion and the consequent activation of the P2Y12 receptor. PAR4-P2Y12 dimer complex recruits β-arrestin and Akt (blue arrows) leading to the activation of integrin and platelet aggregation. On the other hand, TXA2 generation (red arrows) depends on Erk1/2 signaling triggered by the three receptors. TXA2 is involved in platelet recruitment to the site of injury. **B** PAR1-PAR2**.** PAR1 thrombin-induced activation of PAR1 in the early phases of sepsis is vascular disruptive, involving Ca^2+^ mobilization, and Rho/ROCK activity. The inclusion of PAR2 induces a switch to Rac1 signaling and the activation of β-arrestin-dependent ERK1/2 signaling, which is vascular protective. PAR1-PAR2 barrier protection can also depend on FXa. **C** PAR1-PAR3. Thrombin activation of PAR1-PAR3 increases barrier permeability, by stimulating G_α13_ over G_αq_ activity. On this line, the activation of EPCR or PAR3 APC can mediate endothelial barrier protection by directly interacting activating PAR1

In human platelets thrombin activates hPAR1 and hPAR4 (Fig. 1A). However, both human receptors are able to elicit platelet secretion and aggregation, in contrast to the mouse model [19]. In this sense, the precise contribution of each receptor to the dimer in thrombin-stimulated human platelets activation, has been addressed through different approaches [31–33]. PAR4 is a poorly internalized receptor, which accounts for the persistence of its phosphoinositide hydrolysis signal, when compared to that elicited by hPAR1 [32]. In agreement with these results, Covic et al. showed that thrombin-induced Ca^2+^ response has two components: a rapid spike caused by PAR1, followed by a slower prolonged response from PAR4. It is noticeable, that despite its activation rate, which is 20–70-fold slower than that of PAR1, the integrated Ca^2+^ signal generated by PAR4 is greater than the one induced by PAR1 [31]. By using blocking antibodies, antagonists, and desensitization assays, Kahn et al. have shown that PAR1 inhibition blocks platelet activation at low thrombin concentrations (1 nM). In contrast, it only modestly contributes to activation by higher thrombin concentrations (30 nM). More recently, it has been proposed that PAR1-PAR4 heterodimer is needed to ensure rapid onset of the response to thrombin, sustained signalling, and sensitivity concentration within a wide range, since all these three goals would not be reachable with a single PAR receptor. Therefore, the delayed signal triggered by PAR4 serves a different role than the rapid one triggered by PAR1, broadening the time-lapse in which intracellular Ca^2+^ activates the late phase of the platelet aggregation process [34].On the other hand, the activation of both receptors is required for ADP secretion and platelet aggregation at high thrombin concentrations [19]. Even though, PAR4 is much more effective than PAR1 in activating secondary autocrine Ca^2+^ signals from secreted ADP, this response however, requires the activation of PAR1 [31]. It is noticeable that PAR activated ADP secretion activates P2Y12 receptor, which leads to the potentiation of ERK1/2, that generates TXA2 [35]. Additionally, PAR4-P2Y12 dimer complex is required for the recruitment of β-arrestin and Akt which is required for integrin activation [36]. Both pathways are involved in the recruitment of other platelets to the site of injury to reinforce the platelet plug.

The formation of hPAR1-hPAR4 complex has been confirmed in COS7 cells ectopically expressing epitope-tagged receptors, as well as by immunoprecipitation in human platelets [13]. Additionally, PAR1-PAR4 association was also examined by fluorescence resonance energy transfer (FRET) [13]. Furthermore, using BRET, PAR1-PAR4 interface was mapped to transmembrane helix 4 [25]. Mutation of residues near the centre of this helix were sufficient to disrupt the interaction of PAR1 with PAR4 as indicated by a linear BRET curve. These mutants also showed disrupted calcium mobilization in response to PAR4 agonist peptide, implying that if dimer interaction is lost, so will be calcium signalling [26]. PAR4 and P2Y12 specific interaction at the cell surface has been demonstrated using BRET, immunofluorescence microscopy, and co-immunoprecipitation [36].

### PAR1-PAR2 heterodimer

Substantial amount of evidence supports the physiological relevance of PAR1-PAR2 heterodimers, particularly in endothelial cells and in the cardiovascular system in general. In this case, the activation of PAR1 by thrombin, would allow PAR1 tethered ligand domain to bind *in trans*, to trigger PAR2 signaling responses that differ from those elicited by either receptor protomer. The idea that the PAR1 tethered ligand can bind intermolecularly to activate PAR2 *in trans* was tested using synthetic peptide ligands [20]. PAR1 agonist peptide was shown to activate PAR2 with similar potency to the native PAR2 agonist peptide, thus suggesting that either PAR2 can endure a wide range of mutations in critical residues of its ligand site compared to PAR1, which is not the case, or SFLLRN agonist peptide, commonly used to activate PAR1, is also able to activate a neighboring PAR2. Direct association between PAR1 and PAR2 has been confirmed using a variety of methods: PAR1-blocking antibodies [37], FRET imaging, and coimmunoprecipitation using a membrane- impermeable cross-linking agent [14]; and finally, BRET further confirmed these results [38].

PARs have been shown to regulate vascular function in both normal and pathological conditions [2]. PAR1 thrombin-induced activation during early phases of sepsis is generally vascular disruptive, however, as PAR2 expression increases, PAR1 response switches to vascular-protective (Fig. 1B) [14]. It is noticeable that PAR2 expression is increased in endothelial cells by pro-inflammatory mediators, and is considered to play a major role in later stages of sepsis by promoting vascular-protective signaling [2]. Additionally, PAR1 activation triggers Ca^2+^ mobilization, and RhoA activation, which results in endothelial barrier disruption [39]. However, as sepsis progresses, PAR1 signaling switches to Rac1 signaling. This swap is dependent on PAR2 expression and function [14]. In agreement with these findings, PAR2 can increase β-arrestin-dependent ERK1/2 signaling triggered by thrombin-activated PAR1 [38]. β-arrestins are mainly recruited by PAR1 upon activation, and this association is transient. However, PAR2 recruitment renders a stable complex that co-internalizes into endocytic vesicles [38]. The exact signaling mechanisms involved in endothelial cytoprotective signaling, remain elusive. On this line, it is interesting that activation of FXa, which is responsible for the in vivo activation of prothrombin, can mediate barrier protection on endothelial cells by triggering the PAR1-PAR2 dimer to enhance barrier integrity. Although the mechanisms and physiological role of this activation are currently unknown, it is possible that FXa and other agonists of endothelial PAR2 may have a potential protective effect, which should be explored in models of local and systemic inflammation in vivo [40]. In the coronary arteries, PAR1-PAR2 activation induces vascular smooth muscle contraction through a Erk1/2-regulated process, and leads to the release of vasorelaxant agonists, including nitiric oxide, prostanoids and endothelial-derived hyperpolarizing factors [41]. Additionally, PAR1-PAR2 dimer has been shown to stimulate metastasis of human melanoma as well as prostate cancer cells [42, 43].

Shi, et al. proposed that circulating tumour cells generate thrombin, that most likely activate PAR1 in the context of metastasis, and PAR2 transactivation might contribute to metastatic success through enhanced cell motility [43]. Therefore, the cellular context of PAR1-PAR2 dimer appears to be crucial for its function [41].

### PAR3 heterodimers

As described above for PAR1-PAR2 heterodimer, most of research in this area has been aimed to understanding the opposing roles of PARs in endothelial function. Thrombin activation of PAR1-PAR3 can increase barrier permeability, by favouring G_α__13_ activation over G_α__q_ (Fig. 1C) [24]. Therefore, PAR3 is acting as PAR1 allosteric modulator. On the other hand, the anticoagulant protease, activated protein C (aPC) has a cytoprotective role in endothelial cells and neurons [44, 45]. It has been proposed that aPC’s cyto-protective role is mediated by the endothelial protein C receptor (EPCR), which might interact directly with PAR1 [46]. Interestingly, aPC can cleave PAR3, and activate PAR1 via EPCR in endothelial cells triggering barrier protective effects (Fig. 1C) [47]. However, in mouse podocytes, which are non-endothelial cells that lack EPCR, aPC conveys cytoprotection through the cleavage and activation of PAR3 and PAR1 [48]. Specific antibodies directed to PAR1 or PAR3 were found to block the protection from apoptosis by aPC, suggesting that both receptors are required for aPC cytoprotective signaling [48]. The mechanism, however remains elusive, and so far, it is only known that Caveolin 1 dephosphorylation is required [48]. The direct interaction of PAR1 and PAR3 has been assessed in human pulmonary artery endothelial cells and HEK 293T cells, using BRET [24].

As for PAR1-PAR3 heterodimers, Madhusudhan et al. tested PAR2 and PAR3 as potential candidates for mediating aPC-induced cytoprotective signalling in human podocytes [48]. However, unlike their human counterparts, mouse podocytes do not express EPCR and express very low levels of PAR1 and PAR4 (mRNA and protein), as opposed to PAR2 and PAR3, which are readily detectable. aPC shows anti-apoptotic effect in these cells; which can be blocked by both PAR2 and PAR3 agonist peptides or by knocking-down PAR2 and PAR3 in these cells. It is still unclear whether the dimerization between PAR2 and PAR3 depends on transmembrane interactions or if it relies on the donation of its tethered ligand domain. However, these findings confirm the role of PAR2 and PAR3 activity in aPC-induced cytoprotection and demonstrate that PAR3 is able to modulate PAR2 activity, most likely through the formation of a dimer [48].

Additionally, the interaction of PAR2 and PAR3 has been studied in isolated regulatory T-cells, where aPC inhibits T-cell reactivity through the activation of PAR2-PAR3 signaling [49]. Preventing the proteolytic cleavage of PAR3 N-terminal region, abolishes aPC’s effect on allogenic T-cell stimulation. An antibody that inhibits aPC docking to EPCR is not able to block the inhibitory effect of aPC on T-cell activation, therefore discarding it as aPC receptor and suggesting that PAR3 functions as a cofactor of a distinct receptor. To test the role of PAR1 or PAR4, inhibitory peptides that blocked their activation were used, but had no effect on aPC-induced cytoprotection. The role of PAR2 was addressed by knocking-down its expression in primary human T-cells; in this context, aPC signaling was ablated thus confirming PAR2-PAR3 dimer as aPC’s effector [49]. Furthermore, the physiological relevance of this pair has been established, since it has been shown that PAR2-PAR3 cofactoring on T-cells is required for the inhibition of Graft-vs.-host disease mediated by aPC. This new function of aPC-signaling involving PARs in T-cells may be of utmost importance in the design of novel therapies for Graft-vs.-host disease prevention [49].

## PAR-triggered transactivation of other types of receptors

This signaling paradigm was first described for PAR1 and the Epidermal Growth Factor receptor (EGFR), but it has been described for other receptor tyrosine kinases (RTKs) as well as serine/threonine kinase receptors (RSTKs), toll-like receptors (TLRs) or even ion channels [50–52]. It should be noted that even though the direct interaction between PARs and these receptors has not been confirmed in some cases, we will address the interactions and their signaling mechanisms and the physiological importance of their interaction.

### PAR-mediated Receptor Tyrosine Kinases (RTKs) transactivation

**Fig. 2:**
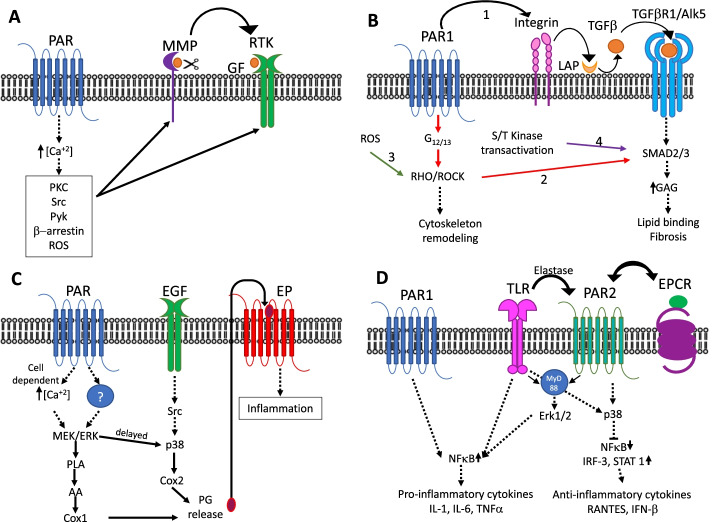
PAR-Triggered transactivation of distinct receptor types. **A** Receptor Tyrosine Kinases (RTKs). PAR-dependent stimulation of RTKs can be triggered, by shedding of RTK ligands, the membrane-bound metalloproteinases (MMP), or by generating downstream signals which activate RTKs on their cytoplasmic domains. **B** Receptor-serine/threonine- kinases (RSTK). TGF-β requirement for PAR1-Alk5 activation, depends on the availability of ligands: 1. PAR1 binding to integrins activates the latency-associated peptide (LAP) on TGF-β1 and -β3 (Black arrows). 2. PAR1 activation of RhoA/Rho kinase (ROCK) pathway promotes the phosphorylation of SMAD2/3 (red arrow). 3. Reactive oxygen species (ROS) induce ROCK signaling (green arrow). 4. Intermediate serine/threonine kinases phosphorylate/activate SMAD2/3 linker region (purple arrow), driving to proteoglycan synthesis and glycosaminoglycan (GAG) gene expression. The activation of these signaling pathways may drive cytoskeleton remodeling, or an increased binding to lipids, leading to fibrosis. **C** Prostanoid receptors. PAR activation stimulates PLA2 activity by two mechanisms: The first involves the elevation of intracellular Ca^2+^, whereas the other is independent of such elevation; since the intermediate that activates this pathway is not known, we have illustrated it with a question mark. Both pathways depend on the activation of MAP kinases. COX-2 induction is mediated by the delayed activation of ERK1/2, p38 MAPK, Src and EGF receptors. PGE2 release is involved in both early, and late phase inflammation. **D** Toll-like receptors (TLRs). PAR2 and TLR4 interaction is mediated by the adaptor protein myeloid differentiation factor 88 (MyD88), or by the release of elastase induced by TLR4. Signaling converges in the induction of NF-κB, and the release of pro-inflammatory cytokines; or in the repression of NF-κB and the up-regulation of IRF-3 and STAT1. This process up-regulates anti-inflammatory cytokines such as RANTES and IFN-β. PAR1 promotes NF-κB activation, whereas PAR2 can promote its activity via ERK1/2 phosphorylation, or down-regulate it via p38

Transactivation of EGFR was first described by Axel Ullrich’s group, in Rat-1 fibroblasts. They reported that EGFR was rapidly tyrosine-phosphorylated in response to GPCR agonists such as endothelin-1, lysophosphatic acid or thrombin, suggesting for the first time, the existence of an intracellular mechanism for RTK transactivation. This finding implied that RTKs could function as downstream mediators in GPCR mitogenic signalling [53]. Later work showed that GPCR-dependent stimulation of the EGFR is triggered by membrane-bound metalloproteinases (MMP), which mediated the shedding of ligands that activate this growth factor receptor. This activation mechanism is also shared by platelet-derived growth factor receptor (PDGFR) and vascular endothelial growth factor receptor (VEGFR) [51]. Transactivation of these RTKs may also derive from the generation of second messengers such as Ca^+2^, PKC, protein tyrosine kinases such as Src and Pyk, β-arrestin or reactive oxygen species (ROS) (Fig. 2A) [51]. PAR activation can transactivate EGFR particularly by signalling from PKC, Src and ERK1/2 [41, 54, 55]. Interestingly, besides triggering a phosphorylation cascade, c-Src may phosphorylate p47phox Nox2 cytosolic subunit, enabling the transactivation of RTKs via ROS [56]. On this line, PDGFR, and VEGFR2 may also be transactivated by ROS [57, 58]. Furthermore, PAR2 and PAR4 promote ROS signalling in the airways and platelets respectively [59, 60], and could also transactivate RTKs through this mechanism.

Noticeably, PAR activation might involve a network including more than one RTK. On this matter, Chandrasekharan and colleagues described a complex signalling interaction in endothelial cells involving PAR1, EGFR and VEGFR [61]. This work showed the synergistic induction of MAP kinase phosphatase-1 by thrombin and EGF through two distinct PAR-1-activated pathways; one dependent on ERK, and a second one requiring VEGF receptor-2 and JNK activation. They demonstrated that VEGFR mechanism of activation does not depend on the secretion of VEGF-A, and therefore discarded the autocrine activation of this receptor, although they failed to co-immunoprecipitate them, further supporting PAR1 role in transactivation [61].

The transactivation of EGFR by PAR1 and PAR2 plays an important role in cancer cell migration, and has been described for lung [62], kidney [63], colon [54, 55, 64] and gastric carcinomas [65, 66]. Additionally, PAR4 is able to transactivate the EGFR and ErbB-2, by a Src-mediated mechanism involving p42/p44 MAPK and p38 MAPK in mouse cardiomyocites [67].

PAR2 transactivation of PDGFR-β has been studied in human monocytes, fibroblasts, and porcine aorta endothelial cells. In this study, tissue factor-factor VIIa (TF/FVIIa) cleavage of PAR2 induces Src-mediated activation of PDGFR [68]. Additionally, in liver carcinoma cells, PAR2 stimulation leads to the transactivation of Hepatocyte growth factor receptor, c-Met, which in turn, promotes cell migration and invasion [69]. Similarly, it has been shown that agonists for PAR1, and PAR4 can also activate PDGFR and c-Met, therefore it has been suggested that these receptors may act in a coordinated fashion to promote the migration of hepatocellular carcinoma cells [70].

### PAR-mediated receptor-serine/threonine- kinase (RSTK) transactivation

Most of the work regarding PAR-induced transactivation has focused on RTKs, however PARs can also stimulate, the canonical SMAD signalling pathway, which, classically is activated by transforming growth factor beta (TGF-β)/activin/ bone morphogenetic protein (BMP) superfamily of growth factor receptors (for review refer to [71]). By this process, PAR cellular responses are widely amplified. It is remarkable that this signalling mechanism accounts for approximately 50% of the total genes regulated by PAR1 with 177 genes regulated, as opposed to 209 activated by PAR1-RTK signalling [52]. Therefore, RSTK transactivation seems to be as relevant as RTK, measured through the ability to stimulate gene expression in array studies [52].

In vascular smooth muscle cells, thrombin treatment has been shown to induce the temporal increase in Smad2C phosphorylation which is blocked by PAR1 antagonists. This effect was prevented by an Alk5 (activin-like kinase 5; TGF-β type I receptor or TGFBR1) inhibitor [72]. It is noticeable, that the requirement for TGF-β on PAR1-Alk5 activation, depends on the cellular context: In vascular smooth muscle cells it is not necessary [72], whereas in in mouse lung epithelial cells [73] and buccal fibroblasts [74] PAR1 can bind to integrins α_v_β_6_, α_v_β_1_, α_v_β_3_, or α_v_β_5_ that interact with the latency-associated peptide (LAP) of TGF-β1 and -β3 to activate latent TGF-β. Interestingly, in these three cell types, thrombin activation leads to the phosphorylation of SMAD2/3, by triggering the RhoA/Rho kinase (ROCK) pathway which is related to the modification of cytoskeletal structures [73–75]. On this regard, it has recently been shown that an increase in reactive oxygen species (ROS) stimulates ROCK signalling pathways that in turn activate TGFBR1 without the activation of other pathways [76]. Therefore, thrombin-induced transactivation of TGFBR1 by ROCK would involve, at least two independent pathways: the activation of G_α12,13_, and/or the generation of ROS (Fig. 2B).

TGF-β-activated SMAD2/3 contains an N-terminal domain, a central linker region, and carboxy-terminal domain, directly phosphorylated by TGFBR1. The linker region can also be phosphorylated by intermediate serine/threonine kinases triggered by other receptors, which links SMAD cascades to these receptors and represents a transactivation target [77]. It is noticeable that thrombin transactivation of EGFR in keratinocytes, leads to the phosphorylation of the Smad2 linker region, and the phosphorylation of this region drives proteoglycan synthesis and glycosaminoglycan (GAG) gene expression [77]. This linker region plays a central role in integrating transactivation-dependent signalling that leads to the modification of GAG chains [75, 77]. These changes in proteoglycan structure occur mainly though the elongation of these chains, which in turn result in an increased binding to lipids in vitro; and this accumulation has been related to the progression of atherosclerosis [77].

PAR2 has also been shown to interact with the TGF-β receptor in primary human proximal tubular epithelial cells [78], and a pancreatic ductal adenocarcinoma cell line [79]. PAR2 and Alk5 have been shown to interact directly (they can be immunoprecipitated) and promote ERK activation independently of C-terminal SMAD2/3 phosphorylation [80]. This pathway drives TGF-β-induced epithelial-to-mesenchymal transition, which is essential for cell transformation in cancer [79]. It has been proposed that TGF-β/ALK5 is the factor responsible for the transactivation of PAR2, since a PAR2-agonist peptide by itself failed to promote TGF-β1-induced C-terminal phosphorylation of Smad3 [81].

The physio-pathological role of PAR transactivation of RSTKs signalling is highlighted by the role its components play in triggering of tissue fibrosis and cancer. Since these pathways converge on the regulation of certain matrix genes, or display mutual regulation of their signalling components, the interactions between PAR1/PAR2 and TGF-β signalling, eventually result in feed-forward loops/vicious cycles of matrix deposition and malignant traits that exacerbate fibrosis and oncogenesis, respectively [82]. As examples of these ailments we can mention, atherosclerosis and cardiac disease [75, 82], oral submucous fibrosis [74], pulmonary fibrosis and lung injury, kidney injury and tubulointerstitial fibrosis [82, 83], arthritis [84], as well as wound healing disorders and cancer [82]. Further study of these interactions will be important for identifying potential therapeutic targets.

### PAR transactivation of prostanoid receptors

PAR signaling directly associated with the activation of prostanoid receptors has been identified in different organs including the airways [85], gastrointestinal system [86], the kidneys [87] and the urogenital tract [88]. The role of prostaglandin E2 (PGE2) is mediated by the activation of the prostanoid receptor (EP) subtypes EP2, EP3 and EP4 [85, 89]. Prostaglandins are derived from arachidonic acid through the action of cyclo-oxygenase (COX), of which two isoforms of have been identified, COX-1 and COX-2 [90]. It should be noted that in this section, the term “transactivation” is used loosely, since it has been shown in platelets that, COX-2 is upregulated in inflammation, and this increase is cycloheximide-sensitive, therefore it depends on protein synthesis, which does not align strictly with the term “transactivation”. However, in most cells and tissues, COX-1 is constitutive and is responsible for the maintenance of PGs physiological homeostasis, whereas the expression of COX-2 is up-regulated in inflammatory cells and inflamed tissue [90]. Although most of the work in this field is focused on thrombin induction of COX-2 [91], the activation of PAR1 and PAR2 triggers PGE2 release by a mechanism involving both, COX-1 and COX-2 in mouse osteoblasts [92], and in the tracheal and bronchial smooth muscle [89]. Phospholipase A2 (PLA2) is responsible for the cleavage of phospholipids containing arachidonic acid, which will in turn activate COX enzymes. It is activated by phosphorylation, generally in response to the elevation of intracellular calcium, but can also be activated by thrombin or tryptase in the absence of calcium [88]. The phosphorylation of cPLA2 has been observed to increase following 5 min stimulation, and this effect is abolished by the MEK inhibitor U0126 [93]. The activation of PLA2 by intracellular Ca^2+^ depends on the type of activated receptor and the cellular context: since PAR1-activated osteoblasts are insensitive to the Ca^+2^ chelator BAPTA/AM, and PKC inhibitors, whereas PAR2-activated lung epithelial cells require this stimulation for transiently activating ERK, and subsequently stimulating PLA2 (Fig. 2C) [89, 92]. On the other hand, as expected, the induction of COX-2 is delayed, and is mediated by ERK1/2, p38 MAPK, Src and EGF receptors (Fig. 2C) [62, 92]. In gastric mucosal epithelial cells, a similar mechanism is observed, where PAR1 agonist peptide caused transient (5 min) or persistent (6 h or more) ERK and p38 MAPK phosphorylation, respectively, followed by delayed reinforcement at 18 h [94].

### PAR1/PAR4—P2Y receptor subtype, P2Y12

As discussed above, PAR4-P2Y12 plays an important role in platelet activation. This complex has been studied in pathological contexts: Acute coronary syndromes (ACS) are usually treated with an antiplatelet therapy consisting of aspirin and a P2Y12 antagonist (ticagrelor, prasugrel, or clopidogrel) for the prevention of pathologic platelet activation and consequent atherothrombotic events. However, the role of other platelet activators such as PARs, which contribute to platelet hyperreactivity, has been poorly addressed. Wadowski et al., found that ticagrelor exerts a more pronounced antiplatelet effect on PAR mediated platelet activation than prasugrel in ACS patients. While platelet activation stimulated by ADP was, in general, very low in these patients, PAR mediated platelet activation was preserved in many patients, and the contribution of this increment in platelet activation to adverse clinical outcomes, remains to be established [95]. However, it should be noted that even though the inhibition of PAR-1 by vorapaxar in addition to antiplatelet therapy, decreased the composite of myocardial infarction, stroke, and cardiovascular death in ACS patients, it significantly increased the risk of bleeding including intracranial haemorrhage, therefore, vorapaxar PAR-1 inhibition may impair cellular hemostasis excessively, at least in conjunction with antiplatelet therapy [96]. Additionally, in a model of experimental cholestatic liver injury, thrombin-mediated PAR4 activation promoted further ADP-driven platelet activation. This would explain why clopidogrel, an inhibitor of P2Y12 receptors, applied for reducing the risk of heart disease and stroke, exacerbates hepatic injury and cholangiofibrosis in this mouse model, and prompts to further study this interaction in tissues besides the cardiovascular system [97].

Furthermore, PAR polymorphisms may contribute to platelet hyperreactivity and may have an unaddressed role in stroke and cardiovascular disease. Whitley, et. al., found that PAR4 rs773902 SNP increases platelet reactivity to thrombin, and this effect requires P2Y12, since P2Y12 antagonist ticagrelor, but not PAR1 inhibitor vorapaxar, blocked the effect of PAR4 polymorphism on thrombin-induced platelet aggregation. These data underline the importance of understanding the physiological effects of receptor transactivation, as well as their polymorphisms in order to improve drug design and treatments [98].

### PAR4-Bradykinin B2 receptor

The kallikrein-kinin system are proteases, which cleave the non-enzymatic cofactor, high-molecular-weight kininogen, to produce bradykinin [99]. Bradykinin is a 9-amino acid peptide which acts via G protein-coupled B1 and B2 bradykinin receptors, which exert proinflammatory and vasodilatory effects on vascular endothelial and vascular smooth muscle cells (VSMCs) [99]. Bradykinin has been shown to induce bronchoconstriction and hypersecretion of mucus in the airways [100], and appears to have beneficial effects on the progression of type 2 diabetes, hypertension, and ischemic renal injury in rodents [101]. Even though kallikreins may cleave PAR1, PAR2 and PAR4 [102], the pro-inflammatory effects of PAR4 activation rely on bradykinin B2 receptor (B2 receptor) [103, 104]. This question has been addressed in a rat paw or a knee joint inflammation model, in which the PAR4-selective agonist peptide AYPGKF-NH2, induces oedema and granulocyte recruitment that is inhibited by the administration of B2 receptor antagonists [103, 104]. Additionally, in dorsal root ganglion neurons, the PAR4 peptide agonist triggers a significant increase in afferent firing during knee rotation in both painful and pain-free conditions, which is blocked by pretreatment with a B2 antagonist. These data confirm PAR4-B2 interaction, and an important role for this complex in the mechano-sensitivity of knee joint afferent fibres [104]. The mechanism involved in this ‘crosstalk’ remains to be elucidated. Additionally, it has been suggested that B2 receptor could also interact with PAR2 [12], since PAR2-triggered visceral hyperalgesia and PAR2-induced scratching can also be inhibited by B2 receptor antagonist HOE-140 [105, 106]. More recent results have shown that both B2 receptor and PAR2 antagonists significantly attenuated kallikrein 8-induced protective actions on a cardiac ischemia/reperfusion injury model, even though this study did not address the interaction of both receptors, and it is possible that this protease could be simultaneously activating both receptors [107].

### PAR- toll-like receptors (TLRs) and NOD-like receptors

Toll-like receptors (TLRs) and NOD-like receptors (NLRs) are pattern-recognition receptors (PRRs) that detect microbial structures (such as lipopolysaccharides (LPS) and lipopeptides) and in turn activate cells of the innate immune system [108, 109]. PAR-TLR interactions, however, involve not only immune system cells, but also epithelial and vascular endothelial cells, thus exerting an important role in the response against infections [110, 111].

PAR-TLR interactions have been documented both via direct (i.e. PAR2-TLR4 coimmunoprecipitation [112]) and indirect (i.e. signal crosstalk) mechanisms in many conditions ranging from lipopolysaccharide effects, to the study of other TLR-activating ligands in vitro and in vivo [110–114]. Treatment with LPS and PAR2-activating peptides, results in a concentration-dependent regulation of cytokines such as Tumor necrosis factor -α, interferon, or interleukin (IL) -4, IL-6, IL-8, IL-10 and IL-13 [113, 114]. PAR2 and TLR4 interaction is mediated by the adaptor protein myeloid differentiation factor 88 (MyD88) [112], the endothelial cell protein C receptor (EPCR) [115], or can result from the release of elastase by TLR4, which is able to cleave and activate PAR2 [116]. Cooperative signaling convergence has also been observed between PAR2 and both TLR2 and TLR3 [113], as well as between PAR1 and TLR2 and TLR4 [111, 117, 118]. It is worth noticing that regardless of the cell type or receptors involved, signaling converges in the nuclear translocation of NF-κB that is required for the expression of most pro-inflammatory cytokines (Fig. 2D) [111–113, 117]. PAR1 promotes NF-κB activation, whereas PAR2 can promote or inhibit its activity [111, 112]. Moreover, ERK1/2 phosphorylation is also induced by PAR1 agonist peptide [111, 118], and can be promoted by PAR2 agonist [111, 119]. On this line, p38, which has been associated with the inhibition of NF-κB is activated by a PAR2 agonist [111]. The differential engagement of downstream adaptor modules by TLRs and PARs, may be responsible for the differences in signaling pathways, as well as proinflammatory or anti-inflammatory roles observed. Activation of the MyD88-dependent pathway mediates early nuclear translocation of NF-κB which is required for the induction of pro-inflammatory cytokines like IL-1, IL-6, and Tumor necrosis factor -α. In contrast, the delayed NF-κB response is related to the activation of transcription factors such as IRF-3 and STAT1, that up-regulate transcription of cytokines such as RANTES and IFN-β (Fig. 2D) [112, 113].

### PAR—ion channels

Upon the breakdown of the blood–brain barrier, thrombin elicits both neuroprotective and neurotoxic activities [50, 120]. Some of PAR1-mediated neuronal effects are thought to depend on its ability to modulate the function of N-methyl-D-aspartate receptor (NMDAR); a glutamate-gated ion channel that plays a critical role in the nervous system, and has been implicated for decades in a wide variety of neurological diseases. NMDAR consist of a tetramer assembled with two NR1(a-b) subunits, and one or two subunits of either NR2(A-D) or one subunit of NR3(A-B). It is important to note that subunit composition of NMDAR is critical to the diversity of functions related with these receptors (for review see Pagano et al. [121]).

Most of the work addressing the modulation of NMDAR by PAR1 has been restricted to the hippocampus [50, 122]. However, PAR1 is expressed throughout the brain on neurons and astrocytes [123], and recently it has been shown to play a role in the modulation of synaptic NMDARs on nigral dopaminergic neurons and in neurons of the solitary tract [124, 125].

PAR1 potentiates NMDAR function in hippocampal neurons, by modulating glutamate availability. This can be achieved by increasing glutamate release from astrocytes [126], and also through the stimulation of neuronal excitability [122, 127], which induces long term potentiation (LTP), implicated in memory formation [128], the generation of epileptic seizures [129] and ischemia [130]. It is noteworthy that PAR2 has also been involved in motivational learning in rats [131]. On the other hand, PAR1 can also impair LTP expression at Schaffer collateral synapses in the hippocampus. In this area, PAR1 induces a rapid and complex structural reorganization of astrocytic processes surrounding excitatory synapses, which is equivalent to increasing the number of transporters in the area, without modifying their protein expression. This restructuration increases glutamate clearance from the synaptic cleft, therefore limiting NMDAR activation [132]. In this sense, stimulation of PAR1 in nigral dopaminergic neurons promotes the endocytosis of NR2B and NR1 subunits, indicating that by regulating subunit internalization PAR1 is also able to limit synaptic NMDARs [124].

The mechanism through which thrombin triggers opposite responses is currently unknown, although concentration may play a major role. In hippocampal neurons, high thrombin concentrations induce an NMDA-dependent, slow onset LTP; whereas low concentrations of thrombin promote a voltage-gated calcium channels/mGluR-5 dependent LTP, which involves intracellular calcium stores, and the transactivation of PAR1 by Endothelial Protein C Receptor (EPCR) [50]. On this line, activated protein C (aPC), EPCR’s ligand, protects mouse cortical neurons from NMDA-induced apoptosis via PAR1 and PAR3 [45, 133]. Moreover, PAR1 modulation of NMDAR is dependent on the subunit composition. Oocytes coinjected with NR1a and either the NR2A or NR2B subunits are potentiated by thrombin activation of PAR1; whereas coexpression of NR2C or NR2D with NR1a, resulted in receptors unaffected by thrombin activation [122]. Similarly, in the solitary tract and dopaminergic neurons, PAR1 agonist peptide is able to modify NR2B components of evoked excitatory postsynaptic currents (EPSCs) [124, 125].

These data suggest that PAR1 modulation of NMDARs varies according to changes in location and cellular populations, and its role in the modulation of neurotransmission and synaptic plasticity can differ depending on the context in which the receptor is expressed.

Besides PAR1, under physiological conditions, PAR2 is also widely expressed in the central nervous system. However, unlike PAR1, PAR2 has been shown to induce long-term depression (LTD) in hippocampal CA1 neurons, that is dependent on NMDAR activation, particularly on NR2B-containing receptors [134]. More recently, it has been shown that PAR2-induced LTD involves Transient Receptor Potential Vanilloid 4 (TRPV4) [135]; the precise mechanism remains unclear. However, in the peripheral nervous system, PAR2 is involved in nociception and hyperalgesia where Transient receptor potential (TRP) V-channels amplify the proinflammatory and hyperalgesic actions of proteases mediated by NMDARs [136]; suggesting that the key to unravel the mechanisms regulating this complex lay in the peripheric nerves.

Transient receptor potential (TRP) ion channels constitute a large family of 28 members, in mammals, that mediate the transmembrane cellular influx of cations, mainly Na^+^ and Ca^2+^ (for review, see: [137]). TRP channel activity can be modulated by many factors such as changes in surrounding temperature, mechanical stimuli, or specific ligands such as capsaicin, products of lipid metabolism, purine nucleotides, or even inorganic ions such as Ca^2+^ or Mg^2+^. PARs may influence TRP channel activation through the activation of the PLC pathway, which generates three metabolites that modulate the activity of these channels: phosphatidylinositol [4, 5] bisphosphate (PIP2), which can act as a ligand; diacylglycerol, which modulates PKC, a major regulator; and Ca^2+^, which can be, either a direct activator, or a modulator through the activation of calmodulin [137]. Additionally, PAR2 can activate pathways, such as, PLA2, tyrosine kinases, or protein kinase A, that mediate TRP -induced inflammatory pain [138, 139]. PAR1 has been shown to interact with TRPC4 and TRPV4 to influence endothelial permeability in the airways and upper gastrointestinal tract [140]. This interaction induces a sustained Ca^2+^ entry, which results in endothelial junction remodelling in vitro, leading to changes in vascular function [140]. Additionally, PAR1, PAR2 and PAR4 are expressed in sensory neurons and can sensitize TRPV1 through the activation of PKCε, therefore causing neurogenic inflammation and hyperalgesia [141, 142]. PAR2 plays a very important role in the promotion of neurogenic inflammation and pain, since it can also excite peripheral nerve terminals and induce the release of substance P and calcitonin gene-related peptide from their central projections within the spinal cord [143]. Interestingly, the use of an alternative PAR2 cleavage site by cathepsin S couples PAR2 to G_α__s_ which does not mobilize intracellular Ca^2+^, but nevertheless it activates TRPV4, which highlights the variety of mechanisms in which these receptors are interconnected [144]. In the skin, PAR2 and PAR4-triggered hypersensitivity are involved in neurogenic inflammation mediated by TRPV1-4, and TRPA1, that leads to a variety of skin conditions; however, the exact mechanisms have yet to be defined (145).

## Conclusions

This review provides a broad overview on the knowledge regarding PAR receptor-receptor interactions, which involves either direct or indirect mechanisms. The wide variety of these receptor interactions and their impact in pathophysiology, highlights the importance of addressing their study not as a single-unit entity, but as a complex network of proteins. As we delve into the study of PARs, it becomes clearer the importance of understanding the mechanisms of transactivation, and will not only provide novel possibilities for developing pharmacological targets, but will also allow us to grasp different perspectives on cell physiology and disease.

## Supplementary Information

**Additional file 1. Table S1**. Summary of the main characteristics of the PAR receptors.

## Data Availability

Not applicable.

## Abbreviations

ACS: Acute coronary syndromes
ADP: Adenosine diphosphate
aPC: Activated protein C
B2 receptor: Bradykinin B2 receptor
BMP: Bone morphogenetic protein
BRET: Bioluminescence resonance energy transfer
cAMP: Cyclic adenosine monophosphate
CFP: Cyan fluorescent protein
COX: Cyclo-oxygenase
ECs: Endothelial cells
EGFR: Epidermal growth factor receptor
EPCR: Endothelial protein C receptor
ERK: Extracellular signal-regulated kinase
FRET: Fluorescence resonance energy transfer
GFP: Green fluorescent protein
GPCR: G protein-coupled receptors
HEK293: Human embryonic kidney 293
IL: Interleukin
KO: Knockout
LPS: Lipopolysaccharides
LTD: Long-term depression
LTP: Long-term potentiation
MMP: Membrane-bound metalloproteinases
NLRs: NOD-like receptors
NMDAR: N-methyl-D-aspartate receptor
PARs: Protease activated receptors
PDGFR: Platelet-derived growth factor receptor
PGE2: Prostaglandin E2
PKC: Protein kinase C
PLA2: Phospholipase A2
PLC: Phospholipase C
Rluc: *Renilla* Luciferase
ROCK: Rho kinase
ROS: Reactive oxygen species
RSTKs: Receptor-serine/threonine-kinases
RTKs: Receptor-tyrosine-kinases
TGF-β: Transforming growth factor beta
TGFBR1: TGF-β type I receptor
TLRs: Toll-like receptors
TRP: Transient receptor potential
TXA2: Thromboxane
VEGFR: Vascular endothelial growth factor
VSMCs: Vascular smooth muscle cells
YFP: Yellow fluorescent protein
