# Supplementary material for: Diversification of PAR signaling through receptor crosstalk
Source: Cell Mol Biol Lett. 2022 Sep 10;27:77. doi: 10.1186/s11658-022-00382-0 (PMC9463773; doi:10.1186/s11658-022-00382-0)
Supplement: Supplementary file 1 [file 11658_2022_382_MOESM1_ESM.docx]

Table 1. Summary of the main characteristics of the PAR receptors.

Signalling pathway	PAR2	Signalling pathway	PAR3	Signalling pathway	PAR4		Reference
Gαq/11/Ca2+, Gα12/13, Gαi/o, β-arrestins	VIIa/Xa		Thrombin	Gαq/11/Ca2+, Gα12/13, Erk1/2	Thrombin	Gαq/11/Ca2+, Gα12/13, Erk1/2	
	VIIa/Xa/TF				Plasmin		
Gαq/11/Ca2+, Gα12/13, β-arrestins, Erk1/2	Trypsin	Gαq/11/Ca2+, Gα12/13, Gαs, β-arrestins, Erk1/2			Trypsin		
β-arrestins, Erk1/2, Akt	Tryptase	Gαq/11/Ca2+, Gα12/13, Gαs/cAMP, β-arrestin, Erk1/2, MAPK, Akt			Cathepsin G		Alberelli [2], Chandrabalan [15]
	MT-SP1				Gingipain-R		
	TMPRSS2						
	APC						
	Acrosin						
Gα12/13, MAPK							
Gα11/Ca2+, Erk1/2							
	Plasmin		Proteinase 3				
Gαq/11/Ca2+, Gα12/13, Gαs/cAMP, β-arrestin, Erk1/2,	Cathepsin G	Gαs/cAMP	Elastase				
Gαs/cAMP	Proteinase 3						Hirano [146]
Gαi/o, MAPK	Elastase	Rho, Erk1/2					
Gαi/o							
	Endothelium, lung fibroblasts, neurons, astrocytes, leukocytes, mast cells, gastrointestinal tract, lung epithelium, airway and vascular smooth muscle cells, keratinocytes, renal tubular cells		Platelets, airway smooth muscle cells		Platelets, megakaryocytes, endothelium, neurons, astrocytes, immune cells		Chandrabalan [15]
	Trachea, liver, pancreas, small intestine, colon, kidney, prostate		Trachea, thymus, heart, pancreas, stomach, small intestine, kidney, lymph node		Thyroid, lung, pancreas, small intestine, colon, placenta, adrenal gland, testis, prostate, uterus, skeletal muscle, lymph node		Chandrabalan [15]
	Proinflammatory, vasodilation, inflammatory cell recruitment, endothelial/epithelial barrier disruption, visceral hypersensitivity, osteophyte formation, profibrotic, apoptosis, cell proliferation, cell migration and invasion, itch, proalgesic, hyperalgesia, hypotension		Cofactor for PAR1, PAR2 and PAR4		Platelet activation/aggregation, apoptosis, neutrophil recruitment,proalgesic, analgesic		Chandrabalan [15]
	Impaired leukocyte migration; impaired allergic inflammation of airway, joints, kidney		Protection against thrombus formation/pulmonary embolism		Protection against thrombus formation/pulmonary embolism		Chandrabalan [15]
